# The Recent Rise of Suicide Mortality in the United States

**DOI:** 10.1146/annurev-publhealth-051920-123206

**Published:** 2021-10-27

**Authors:** Gonzalo Martínez-Alés, Tammy Jiang, Katherine M. Keyes, Jaimie L. Gradus

**Affiliations:** 1 Department of Epidemiology, Mailman School of Public Health, Columbia University, New York, NY, USA; 2 Department of Psychiatry, La Paz University Hospital, Madrid, Spain; 3 Centro de Investigación Biomédica en Red de Salud Mental (CIBERSAM), Instituto de Salud Carlos III, Madrid, Spain; 4 Department of Epidemiology, School of Public Health, Boston University, Boston, Massachusetts, USA; 5 Department of Psychiatry, School of Medicine, Boston University, Boston, Massachusetts, USA

**Keywords:** suicide, self-injurious behavior, suicide rates, trends

## Abstract

Suicide is a major public health concern in the United States. Between 2000 and 2018, US suicide rates increased by 35%, contributing to the stagnation and subsequent decrease in US life expectancy. During 2019, suicide declined modestly, mostly owing to slight reductions in suicides among Whites. Suicide rates, however, continued to increase or remained stable among all other racial/ethnic groups, and little is known about recent suicide trends among other vulnerable groups. This article (*a*) summarizes US suicide mortality trends over the twentieth and early twenty-first centuries, (*b*) reviews potential group-level causes of increased suicide risk among subpopulations characterized by markers of vulnerability to suicide, and (*c*) advocates for combining recent advances in population-based suicide prevention with a socially conscious perspective that captures the social, economic, and political contexts in which suicide risk unfolds over the life course of vulnerable individuals.

## 1. INTRODUCTION

Suicide is the leading cause of violent death worldwide and accounts for 1.5% of global mortality (127). Death by suicide has a profound impact on society, affecting families and communities over generations. Globally, suicide mortality has decreased over the last three decades (85). In the United States, however, suicide rates increased by 35% between 2000 and 2018 (52, 114), contributing notably to the stagnation and subsequent decrease in US life expectancy (134). US suicide rates decreased modestly in 2019 (113), generating a new atmosphere of guarded optimism. This decline was due primarily to a slight decrease in the suicide rate among White persons. During this time, suicide mortality increased or remained stable among all other racial/ethnic groups, and little is known about recent suicide trends among other vulnerable groups. Accordingly, suicide in the United States constitutes a major public health crisis, in urgent need of solutions.

This article summarizes suicide trends in the United States and reviews the existing evidence on the causes underlying the recent rise of suicide mortality. We begin by reviewing US suicide mortality trends over the twentieth and early twenty-first centuries. We then review potential group-level causes of increased suicide risk among subpopulations characterized by specific markers of vulnerability to suicide; these markers include geography, age, race and ethnicity, sexual and gender minoritization, military membership, and incarceration. Next, we discuss recent US suicide mortality trends in the global context. We conclude by looking into the future, urging researchers and decision makers to couple recent advances in population-based suicide prevention with a socially conscious perspective that captures the social, economic, and political context where suicide risk unfolds over the life course of vulnerable individuals. Throughout this review, we focus on suicide mortality specifically. Nonfatal suicidal behaviors have unique trends and risk markers, and a detailed discussion of these differences is beyond the scope of the current manuscript.

## 2. TRENDS IN SUICIDE IN THE UNITED STATES

Information on suicide mortality in the United States before 1900 is scarce, consisting of suicide counts for only a few states and years (124), and cannot be compared with data based on modern registration standards, which were established in the early twentieth century. Between 1905 and 1914, age-standardized suicide mortality in the United States was roughly stable at ~ 18–21 suicides per 100,000 (including all age groups and standardized to the 2000 US standard population). During World War I, suicide decreased in the United States, reaching a minimum rate of 13.4 suicides per 100,000 in 1920 (25). This decrease was followed by a marked increase, further accelerated during the Great Depression, leading to a historical peak of 21.9 suicides per 100,000 in 1932—with the highest suicide rates among adults aged 55–74 years—at ~50 suicides per 100,000. Suicide rates then decreased sustainedly until the end of World War II, across all age groups but especially among those aged 45–74 years. By 1942, the highest suicide rates affected individuals aged 75 years or older, a pattern that lasted until the twenty-first century (73). Between 1945 and 1992, US suicide mortality remained roughly stable between 12 and 14 deaths per 100,000, with notable dips during the early 1950s and 1980s and a subtle increasing trend over the 1960s and 1970s (110). Rates between 1945 and 1992, however, evolved heterogeneously across age groups: Suicide mortality decreased among individuals aged >54, did not change substantially among individuals aged 35–54, and increased among individuals aged 15–34, with patterns suggesting the presence of both period and cohort effects (73). Suicide mortality then dropped markedly over the 1990s, across sex, age groups, and races/ethnicities, reaching a historical low of 10.4 deaths per 100,000 in 2000 (76).

**Figure 1:**
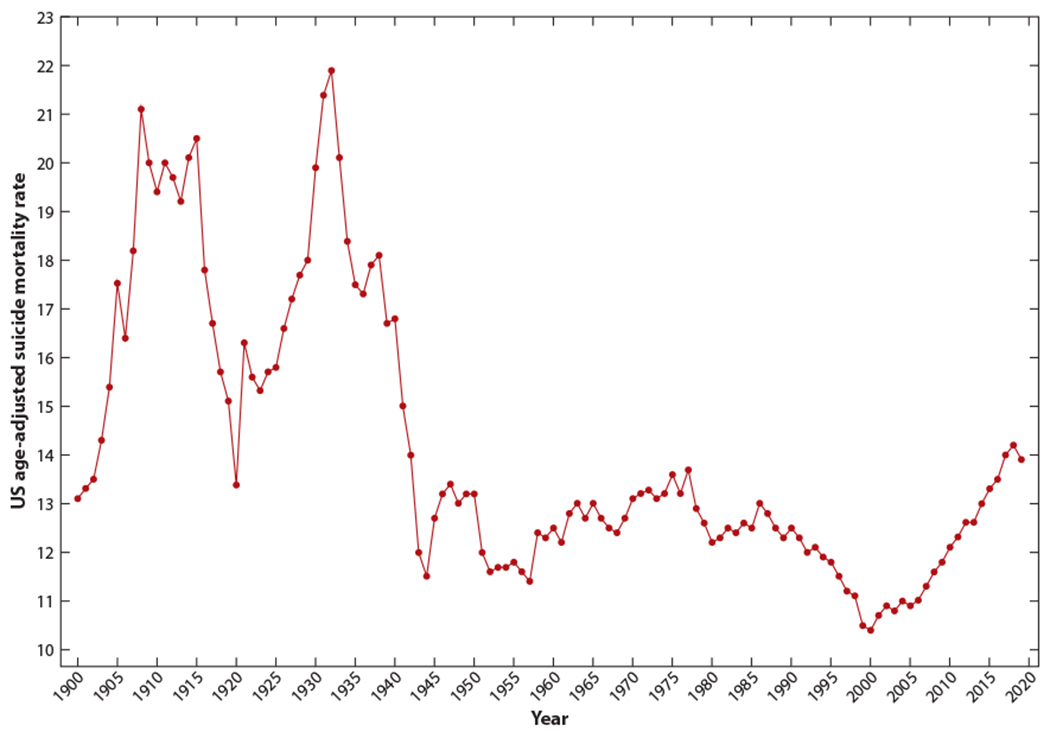
US suicide mortality trends between 1900 and 2019, age-adjusted to the 2000 US population.

After 2000, suicide rates in the United States entered a two-decade phase of uninterrupted increase that affected 44 of 50 US states and every sociodemographic group, though the largest increases were among men aged 45–64, in rural areas, and for suicide by methods other than firearm (114). Between 2000 and 2018, suicide mortality escalated by 35%, with the suicide rate at 14.5 per 100,000 people in 2018, the highest rate in more than 50 years (Figure 1). As a result, suicide is currently the second leading cause of death among adolescents and young adults and the tenth leading cause of death overall (50).

In 2019, US suicide mortality risk declined by 2.1% compared with risk levels in 2018 (113), a decline that continued in 2020 according to preliminary data (37). Notably, this change was due to declines in firearm suicide among White men and suicide by suffocation among White women; suicide mortality remained unchanged or increased slightly among all other races/ethnicities (113). The variation in suicide mortality was also heterogeneous across age, levels of urbanization, and geographic areas. For instance, suicide rates increased in roughly 1 in 3 US states, including several states where rates were already among the highest nationally, such as Wyoming, Alaska, Montana, and South Dakota.

Measuring suicide mortality is inherently challenging because it requires judgment of the deceased’s intentionality. Accordingly, suicide rate estimates based on mortality statistics may be underestimated (118, 131). Furthermore, the extent to which mortality statistics may inaccurately capture true suicide mortality rates is related to the frequency of other mortality causes that are prone to conflation with suicide. For example, in the United States, there has been an ongoing drug overdose epidemic, and determining the intent behind lethal overdoses (as intentional or unintentional) can be difficult. Reported suicide rates must always be interpreted with this critical context in mind.

## 3. MARKERS OF SUICIDE VULNERABILITY IN THE UNITED STATES

Although recent declines in suicide mortality may generate some optimism, they also obscure the dynamics of suicide mortality of continued concern among specific vulnerable groups. Monitoring deviations from the national trend in suicide mortality is paramount for suicide prevention because it can guide identification of vulnerable groups, generation of causal hypotheses, targeting of modifiable risk factors, and prioritization of interventions. In this section, we review salient markers of vulnerability to suicide in the United States, including geography, age, race and ethnicity, sexual and gender minoritization, military membership, and incarceration. In the following section, we include a detailed discussion of the causes that may underlie these trends.

### 3.1. Geography

Suicide mortality in the United States follows a marked geographical pattern (25). The highest suicide rates (e.g., above 15 deaths per 100,000) can be found in the West, Midwest, and South. In Western states, only California has a suicide rate below the national average, and rates are especially high in Mountain Division states such as Wyoming and Montana (29.3 and 26.2 suicides per 100,000 in 2019, respectively). Most Midwestern states from the West North Central Division (e.g., South Dakota, with 20.9 deaths per 100,000 in 2019) and some Southern states (e.g., Oklahoma, with 20.5 deaths per 100,000 in 2019) also have rates markedly above the national average. Eastern states traditionally have the lowest suicide rates in the country, with some exceptions in New England (e.g., Maine, with 19.4 suicides per 100,000 in 2019). Overall, rural counties have markedly higher suicide rates, compared with urban ones, and have also experienced greater increases in suicide mortality over the last two decades (87).

### 3.2. Age

Suicide mortality typically has a first peak among young adults and a second peak (of greater magnitude overall) among older adults, especially among older men. Between 1999 and 2017, rates were highest for women aged 45–64 and men aged 75 and over, compared with other ages. Furthermore, over the same period, suicide mortality in the United States increased for all age groups except those aged 75 and over (50, 52). Women in their sixties and men in their fifties experienced the largest increases overall, suggesting the presence of a cohort effect (e.g., an effect resulting from the exposures shared by individuals born into a specific historical context) among Baby Boom cohorts, born between 1945 and 1964 (96). The recent increases in suicide have been especially salient for those aged 15–24 years (50): Between 1999 and 2018, suicide rates went up from 3 to 5.8 per 100,000 among women, and from 16.8 to 22.7 per 100,000 among men. This observation, coupled with the fact that suicide rates among individuals aged 15–24 years in the United States have been on the rise for the majority of the last 50 years (76), has generated substantial concern and attention. Though reasons underlying this phenomenon remain largely unknown, recent evidence indicates a presence of suicide birth cohort effects among individuals born after 1990, especially for racial minority populations (75).

### 3.3. Race and Ethnic Group

There are important disparities in suicide rates between racial/ethnic groups as well as differential time trends in suicide rates across racial/ethnic groups in the United States. Non-Hispanic American Indian or Alaska Native (AIAN) persons have the highest race/ethnicity-specific suicide mortality rates in the United States. For instance, in 2017, national suicide rates were 22.4 and 6.1 per 100,000 for men and women, respectively, yet suicide rates among AIAN men and women were 33.8 and 11.0 per 100,000, respectively (29). Moreover, AIAN persons also experienced the largest increases in suicide mortality between 1999 and 2017 across all racial/ethnic groups: 71% in AIAN men and 139% in AIAN women, roughly three times the increase of the overall US suicide rate (51).

Non-Hispanic White persons have the next highest suicide rate in the United States, with 2017 suicide mortality rates of 28.2 and 7.9 per 100,000 for men and women, respectively (29). Also, suicide rates increased by approximately 40% in non-Hispanic White men and 68% in non-Hispanic White women between 1999 and 2017 (29), largely due to increases in suicides in middle-aged and older non-Hispanic White persons. Between 1999 and 2017, suicide rates increased among non-Hispanic White men and women aged 45–64 years, respectively, from 23.4 to 38.2 per 100,000 (a 63% increase) and from 7.0 to 12.8 per 100,000 (an 83% increase) (29).

The suicide rate among Asian and Pacific Islander (API) persons in 2017 (9.9 and 3.9 per 100,000 in men and women, respectively) was lower than the overall US suicide rate. Between 1999 and 2017, rates increased by 10% in API men and 15% in API women (29), also slightly below the national average. Disaggregating the broad API grouping reveals ethnic variation in suicide rates. In 2010, Korean American and Japanese American men had higher suicide rates (19.9 and 15.7 per 100,000, respectively) than did other Asian men (e.g., Chinese American men) (65). Likewise, Korean American and Japanese American women had higher suicide rates (8.1 and 5.0 per 100,000) than did other Asian women (65). Furthermore, there are differences in suicide time trends by ethnic group. Between 2000 and 2010, the suicide rate increased by 72% in Korean American men, 14% in Japanese American men, and 8.2% in Vietnamese American men (65). For women in the same time period, the suicide rate increased by 108% in Korean American women, 61% in Vietnamese American women, and 16% in Japanese American women (65).

In 2019, suicide was the third leading cause of death among Black persons between 15 and 24 years of age (23). Overall, suicide rates among Black persons are lower than the national average, a phenomenon often referred to as paradoxical, given their social, political, and economic disadvantage (103). The gap in suicide rates may be partially explained by the greater likelihood of undercounting of suicide deaths in this group (103, 130). Black persons who die by suicide are less likely than Whites to leave a suicide note and to have a record of mental disorders due to poorer access to health care (56, 103). Between 1999 and 2017, suicide rates increased among Black men and women, from 10.5 to 11.4 per 100,000 and from 1.7 to 2.8 per 100,000, respectively. It is worth noting that the Centers for Disease Control and Prevention (CDC) defines individuals who have origins in any of the Black racial groups of Africa, including immigrants from the Caribbean, South America, and Latin America, as Black persons. Most studies do not disaggregate suicide rates for Black persons, and as a result, differences in suicide rates within this diverse group remain unclear.

Hispanics are the largest ethnic minoritized group in the United States, comprising approximately 18% of the US population. Even though US Hispanics have historically had relatively low suicide rates, suicide was the second leading cause of death among Hispanic persons between 15 and 34 years of age in 2019 (23). Suicide risk among Hispanics in the United States has steadily increased over the last two decades. Among Hispanic men, the suicide rate increased from 10.3 per 100,000 in 1999 to 11.2 per 100,000 in 2017. The suicide rate in Hispanic women increased from 1.9 per 100,000 to 2.6 per 100,000 during the same time period. Of note, Hispanic is defined by the CDC as a person of Cuban, Mexican, Puerto Rican, South or Central American, or other Hispanic culture or origin, regardless of race (5). Despite ethnic variation, studies often do not disaggregate suicide rates by ethnic subgroup, and thus little is known about how suicide rates may differ within Hispanic Americans.

### 3.4. Sexual and Gender Minoritization

Sexual and gender minoritized groups are at a high risk of suicidal behavior. There are limited surveillance data on the suicide rate among sexual and gender minoritized groups in the general US population, despite high rates of suicidal behavior among these populations. For example, lesbian, gay, bisexual, or questioning teens were more than three times as likely to attempt suicide compared with heterosexual students in the United States in 2017 (99). In addition, evidence from the Netherlands shows that suicide risk in transgender persons is higher than that of the general population, underscoring the importance of surveillance of suicide mortality in sexual and gender minoritized groups in the United States (133). Most of the US studies of the association between sexual or gender minority status and suicide have been conducted within the US veteran population, with suicide being the fifth leading cause of death among sexual minoritized veterans in 2017. Among these veterans, the suicide rate increased from 60.9 per 100,000 person-years in 2013 to 81.7 per 100,000 person-years in 2017. There are also sex differences in suicide rates for sexual minority veterans; the age-adjusted suicide rate was 100.1 per 100,000 person-years in male sexual minority veterans and 49.3 per 100,000 person-years in female sexual minority veterans, respectively.

### 3.5. Military Membership

Although the military population has historically had lower suicide rates than the general US population, military suicide rates have been increasing over the past decade (4, 34, 53, 101, 107). The crude military suicide rate appears to be higher than that of the general US population, but the military population is younger and comprises more men than the civilian population (32). After adjusting for age and sex differences, the military’s suicide rates are comparable to or lower than those of the general US population. Specifically, the 2019 suicide rates in Active Component and National Guard members were comparable to the suicide rate in the general US population, while the Reserve (across all components) suicide rate is lower. Similar to the trend of increasing suicide rates in the general US population, suicide rates in the military have also increased over time. Between 2014 and 2019, the suicide rate in Active Component members increased from 20.4 to 25.9 suicide cases per 100,000 service members (32). This increase is attributable to an increase in the suicide rate across all Services. However, the Reserve and National Guard suicide rates did not show evidence of an increase or decrease from 2014 to 2019 (32). The 2019 suicide rate was 18.2 per 100,000 for Reserve members and 20.3 per 100,000 for National Guard members. Suicide decedents were primarily enlisted, male, and younger than 30 years of age, regardless of military population (32).

### 3.6. Imprisoned Persons

Suicide rates among persons involved in corrections are far higher than the national suicide rate, despite important reductions during the 1980s and 1990s: Suicide rates in jails decreased from roughly 130 per 100,000 in 1983 to 47 per 100,000 in 2002 (84). This decrease may have been driven largely by the progressive deployment of mandatory suicide prevention programs in corrections as part of an overall improvement in health care and living conditions in jails and prisons sparked in the early 1980s by civil rights activists (47); the decrease in suicide rate also coincided with reductions in rates of inmate homicide. However, inmate suicide increased between 2009 and 2019, in parallel with the overall national trends. Inmate suicide is more frequent among men than women and among White than Black/African American or Hispanic/Latino individuals (90). The higher suicide risk of imprisoned individuals can be considered just a part of the overall adverse impact that the criminal legal system has had on individuals and communities (59). Suicide rates are threefold higher in jails in comparison rates to prisons. This phenomenon has traditionally been explained using the notion of “shock of confinement” (90, 132, 137). Short, first-time convictions entail substantial acute psychological distress and hopelessness, driven largely by the emotional and socioeconomic impacts of imprisonment (e.g., job or housing loss and destructuring of family). In fact, suicide rates in local jails are about three times higher for nonconvicted than convicted individuals (90).

## 4. CAUSES OF TRENDS IN SUICIDE

Suicide is a complex phenomenon, influenced by an interaction between protective and risk factors over time and development. Understanding the causes of suicide among the vulnerable groups described above is critical to suicide prevention. Suicide risk has been conceptualized using a variety of causal frameworks, including biological (92), sociological (33), and psychological (125) theories. From an eco-epidemiological perspective (115), the etiology of suicide is considered multilevel and multicausal, with biological and social causes acting at the individual (i.e., molecular, behavioral) and group (i.e., community) levels and interacting over the life course. Individual-level causes of suicide [e.g., psychiatric diagnoses, substance use disorders, family history of suicide, social adversity (i.e., bankruptcy or loneliness), physical illness, and cognitive problems (40)] have been the subject of extensive research. Because the focus of this article is suicide trends, we focus our discussion on group-level causes of suicide risk, with mention of impact on individual-level factors where relevant.

### 4.1. Access to Means

Access to lethal means plays a critical role in suicide mortality (8, 43). Recent global decreases in suicide mortality are due largely to reductions in access to pesticides in China and India, the two most populated countries, in the context of urban transitions (85). In the United States, where close to 50% of firearm deaths are suicide and nearly 1 in 2 suicides involves the use of a firearm, the role of firearms in suicide trends has been the subject of extensive research. A large body of well-designed research (79, 80, 116) indicates that firearm ownership is associated with higher risk of death by firearm suicide at the state, household, and individual levels across sociodemographic groups.

Data from large surveys in the United States indicate that the firearm ownership rate remained roughly stable or slightly decreased between 1999 and 2018 (41), while firearm suicide increased across age groups, especially after 2007, in a pattern indicating a clear period effect. Moreover, recent studies indicate that (*a*) most of the increase in suicide among very recently born cohorts was driven mostly by nonfirearm means (75) and (*b*) firearm ownership rates were not associated with increases in firearm suicide among these specific birth cohorts (74). This evidence suggests that changes in firearm ownership did not play a major role in the most recent increases in suicide rates, including firearm suicide, and instead links these increases to less specific threats to health (e.g., the 2008 economic recession or the opioid overdose epidemic). Notably, access to firearms remains the most important actionable public health target for firearm suicide prevention efforts. Even though recent stable firearm ownership rates seem to be unrelated to contemporary risk increases in youth suicide, long-standing evidence indicates that changes in firearm availability are associated with changes in firearm suicide, especially among youth (79). This evidence, coupled with the fact that firearm sales went up markedly during the initial months of the coronavirus disease 2019 (COVID-19) pandemic (68), especially among purchasers who had previously reported suicidal ideation (2), should generate concern over potential forthcoming increases in firearm suicide mortality.

### 4.2. Structural Racism

Structural racism refers to the ways in which societies foster racial discrimination through mutually reinforcing inequitable systems (in housing, education, employment, earnings, benefits, credit, media, health care, and criminal justice) (7). These systems reinforce discriminatory beliefs, values, and distribution of resources (7), which affect the risk of negative health outcomes, including suicide. High suicide rates among AIAN persons may be attributable to their experience of historical disenfranchisement through genocide, institutional racism, and social, political, and economic oppression (19). Chronic trauma and historical unresolved grief across generations has resulted in poorer socioeconomic and health outcomes, including poverty, unemployment, depression, substance use disorders, and diabetes (18, 19, 117), which may all contribute to suicide risk. The sharp increase in suicide rates in AIAN women may be influenced by the higher level of violence experienced compared with other US women. According to data from a nationally representative sample assessed in 2010, approximately 84% of AIAN women experienced any violence in their lifetime, 56% experienced sexual violence, 56% experienced physical violence by an intimate partner, and 66% experienced psychological aggression by an intimate partner (104). The majority of female AIAN survivors experienced violence by a perpetrator who was not an AIAN person (97%) (104). Yet, federally recognized tribes did not have the authority to criminally prosecute non-Indian offenders until the Violence Against Women Reauthorization Act of 2013 was passed (Pub. L. 113–4). Centuries of oppression and violence against AIAN persons have contributed to the present-day high suicide rates in this population.

Recent findings indicating the presence of birth cohort effects in suicide among Black persons born after the 1970s (75) support a link between criminality, heavy policing, mass incarceration, and suicide; this finding is consistent with evidence of the harmful effects of these factors in many other health outcomes for Black persons (59). A study of predominantly African Americans between the ages of 18 and 24 found that their biggest concerns were aggressive policing, high levels of community violence, and housing instability, which may contribute to high levels of threat, fear, and hopelessness (35). Also, increased visibility of and media exposure to police killings of Black persons have adverse effects on the mental health of Black adults in the general population (15). Notably, as mentioned above, suicide rates are lower among Black persons compared with those in other racial/ethnic groups, which has been described as a “paradox,” given Black people’s burden of cumulative disadvantage through slavery, segregation, and racism, along with associated high rates of morbidity and mortality (103). This paradoxical gap in suicide rates may be partially explained by the greater likelihood of undercounting of suicide deaths in this group (103, 130). For instance, Black persons who die by suicide are less likely than Whites to leave a suicide note or have record of mental disorders owing to poorer access to care (56, 103).

Discrimination, ethnic marginalization, acculturative stress, and economic oppression may contribute to suicide risk among Asian and Hispanic Americans who may share the immigrant experience (135). Structural forces linked with racism, ethnic discrimination, and xenophobia, as well as limited levels of wealth and earnings, have propagated unemployment and job losses among some Hispanic and Asian Americans in the United States, especially during the COVID-19 pandemic (10, 122). In addition, income inequality is rising the most rapidly among Asian Americans compared with any other US racial/ethnic group (63). Disparities in income among API persons may be driven by variation in education, skills, English-language proficiency, and immigration patterns (63). Income inequality may have implications for those in lower socioeconomic groups in terms of reduced economic opportunity and less political influence (63). Moreover, perceived racial discrimination is associated with increased psychological distress, anxiety, and depression among Asian and Hispanic Americans, which may be mediators on the pathway from racial discrimination to suicide (57, 66). One study found that when disaggregating suicide rates in Hispanic persons by immigrant status, the influence of cultural assimilation appeared to be consistent in immigrant and native-born populations (128). Cultural assimilation may contribute to suicide risk by weakening protective factors, including shared belief systems, rituals, and social networks that promote integration and solidarity within ethnic communities (128). As a result, increased isolation and alienation may contribute to suicide risk.

A successful suicide prevention agenda must dismantle structural racism and repair its harms and may be informed by an intersectional approach that considers the intertwined social and power structures related to race and mental health (58).

### 4.3. Economic Factors

Economic uncertainty and financial strain are major determinants of suicide risk at the individual and population levels. A large body of evidence indicates that persons without housing (6, 28, 39) or employment (12, 69) and people facing various other forms of financial strain, such as personal debt (77), financial loss (121), or low income (36), are at a higher risk of suicide. Economic recessions and suicide mortality rates are strongly associated in high-income countries across the globe (9, 27, 30, 93, 98). Notably, this association may be mediated by increases in (especially long-term) unemployment rates (26) and can be moderated by the presence of social welfare protection policies (91). Accordingly, substantial attention has been directed toward the potential link between recent economic recessions in the United States (e.g., the early 2000s recession and the Great Recession of 2008) and the 1999–2018 national increases in suicide mortality.

Initial assessments reported a clear association between the 2008 economic recession and increases in suicide in the United States (100). However, later analyses accounting for pre-existing trends, seasonal patterning, and cross-gender heterogeneity in suicide rates indicated little evidence of an association between the Great Recession and suicide mortality (48), highlighting that the lack of an overall effect may hide considerable heterogeneity across socioeconomic status, age group, and gender. The strength of a potential association between economic recessions and suicide mortality in the United States varies markedly across states (95) and counties (61) and seems to be mediated by group-level unemployment rates (95), in line with other high-income countries, as well as by poverty (61).

Several other economic indicators have been proposed to explain the economic roots of increases in US suicide mortality trends from 1999 to 2018. For instance, income and wealth inequality, which have increased consistently in the United States over the last several decades (with growth in income and wealth tilting markedly to upper-income households), may be salient determinants of suicide risk (71, 78), although the intensity of the association between inequality and suicide seems to vary over time and by region, somewhat hindering our ability to draw general conclusions (119).

As mentioned before, US rural areas have higher suicide mortality rates and have experienced higher increases in suicide trends than have urban ones (87). Trends in economic factors, such as GDP growth, have also diverged by urbanization level over the last few decades, especially in the Western United States, with rural areas showing around 25% reductions in economic growth following the Great Recession, in comparison with urban areas (64). It seems plausible that, while evidence of an overall association between recent economic trends, and especially the Great Recession, and suicide mortality risk seems mixed for the whole United States, increases in income and wealth inequality driving higher poverty rates in rural areas may partially explain these areas’ higher suicide mortality rates and increases in suicide trends.

### 4.4. Opioid Epidemic

The opioid epidemic in the United States may have contributed to the increase in suicide rates over the last few decades. The proportion of suicides that were due to intentional overdose involving opioids increased from 0.75% in 2000 to 3.6% in 2017 (13). In the early 2000s, opioids were increasingly used to treat acute and chronic pain in the United States, fueled largely by new product formulations and aggressive marketing and dissemination efforts by large opioid manufacturing and distribution companies (46, 120, 126). The opioid dispensing rate increased from 72.4 per 100 persons in 2006 to 81.3 per 100 persons in 2012. The overall national opioid dispensing rate decreased between 2012 and 2019, and in 2019, the dispensing rate was the lowest it had been in the last 14 years at 46.7 prescriptions per 100 persons (i.e., more than 153 million opioid prescriptions total) (22). In addition, there has been an increase in the use of heroin and illicitly manufactured synthetic opioids (120). The increased availability of opioids contributed to increased nonmedical opioid use and opioid use disorders, which may be means of suicide. The annual age-adjusted suicide rate with opioid poisoning listed as a contributing cause of death increased from 0.3 per 100,000 in 1999 to 0.7 per 100,000 in 2009 and remained at 0.6–0.7 per 100,000 through 2014 (16). The suicide rate with poisoning listed as a contributing cause of death was the highest among individuals aged 45–54 years between 1999 and 2011 (16), which could be explained by the deaths of despair hypothesis. This notion posits that widening income inequality and productivity slowdown may cause people to feel despair and to resort to coping through the use of opioids, which were made widely available during this time period (21).

### 4.5. Loneliness and Social Isolation

Loneliness (a subjective feeling of being isolated) and social isolation (the objective state of having few social relationships or infrequent social contact with others) are public health problems that may contribute to adverse health outcomes, including suicide (86). Although loneliness and social isolation are not systematically and routinely assessed in the United States, there are potential indicators of their trends. For example, loneliness and social isolation may be on the rise owing to increased population aging, decreased average household size over the last decade in the United States, and a rising number of persons living alone (55, 123). In addition, loneliness and suicide risk may be greater in scarcely populated states, though recent research has yielded mixed results. Mullen and colleagues (83) did not observe differences in loneliness between rural and urban primary care patients, whereas Henning-Smith and colleagues (54) found that persons living in areas with fewer than 10,000 people reported having more family members they could count on and more friends than did urban residents. Furthermore, loneliness levels appear to be greater among non-Hispanic Black residents of rural areas compared with those living in metropolitan areas (54). A 2020 report from the National Academies of Sciences, Engineering, and Medicine provides a comprehensive review of the prevalence of social isolation and loneliness in various populations and their impact on mortality and morbidity (86); groups that are at high risk of loneliness and social isolation include older adults; immigrants; lesbian, gay, bisexual, and transgender (LGBT) populations; racially and ethnically minoritized groups; and victims of elder abuse. Older adults are at particularly high risk for loneliness and social isolation because they are more likely to live alone, to experience loss of family or friends, and to have chronic illness and sensory impairments. Approximately 24% of Americans aged 65 and older are considered to be socially isolated, and 35% of adults aged 45 and older reported feeling lonely (86). Immigrants may experience language barriers and differences in community that influence their social ties. Similarly, LGBT populations may experience more loneliness than their heterosexual peers owing to stigma, discrimination, and barriers to care (24).

## 5. US SUICIDE RATES IN A GLOBAL CONTEXT

It is difficult to directly compare the US suicide rate to that of other countries. Suicide mortality rates and trends (and quality of detection and registration methods for suicide deaths) are highly heterogeneous within and across countries, as originally documented at the end of the nineteenth century by Durkheim’s original observations (33). A recent review identified increasing suicide trends in only 8 out of 195 countries: Zimbabwe, Uganda, Liberia, Cameroon, Jamaica, Mexico, Paraguay, and the United States (85). Global mortality due to suicide decreased by more than 30% between 1990 and 2016, due mostly to declines in suicide mortality in largely populated countries such as India and especially China (85).

Although we can infer from these data that suicide rates have been increasing in the United States in a way that has not been found in most of the rest of the world, there are many potential etiologic and methodologic explanations for these differences, and no studies have included direct international comparisons to identify the likely complex constellation of factors that have generated these observed differences. The preceding material describes factors that may be contributing to the increase in US suicide rates, and it is beyond the scope of this review to describe the impact of each of these factors in other countries and how it may differ. More large-scale research will be needed to fully understand cross-national differences and their impact on suicide rates.

## 6. CONCLUSION

Suicide has a considerable impact on individuals, families, and communities in the United States, resulting in approximately $100 billion in total (direct plus indirect) costs (109). Despite recent slight declines in suicide mortality, rates in the United States have remained at their peak since the early 1940s, and there is reason for concern about suicide trends in specific vulnerable groups, as defined by geography, age, race and ethnicity, sexual and gender minoritization, military membership, and incarceration.

Notwithstanding, substantial advances in suicide prevention have been achieved over the last two decades. Three strategies stand out for their implications from a population-level suicide prevention standpoint. First, strategies to limit access to lethal means (45, 136), such as pesticides (44), unprotected elevated buildings and bridges (11), highly toxic medications (49), or firearms (3, 72), lower suicide mortality risk. Even though rapid reductions in firearm ownership do not seem likely in the United States for historical, political, social, and legal reasons (1, 14, 60), increasing evidence supports alternative methods to reduce access to firearms during suicidal crises. For instance, safe firearm storage interventions (31, 82, 108) have been designed, implemented (106, 112), and trialed (81) and are included in current evidence-based suicide-specific counseling programs (67, 105, 111). Also, red-flag and seizure laws—which allow for temporary prohibitions of firearm purchase or possession (https://www.ny.gov/programs/red-flag-gun-protection-law)—seem effective (62) and are supported by the majority of the US population (94).

Second, media coverage of suicide death as well as high-profile fictional representations of suicide increase the risk of suicide death in the population, especially when they include graphic depictions or prolonged discussions of suicide methods, or the mental health of those who died, or describe suicide as a solution to a problem. In the United States, increases in suicide have been documented following the death by suicide of a popular comedian in 2013 (38) and, though results remains equivocal, the release of a controversial TV series (89), among others. These media effects, however, are not inevitable, and protective “Papageno effects” (88) have also been observed, whereby media reporting prevents the spread of suicidal behavior. Notably, protective efforts based on safe communication patterns, such as mass media (97) and especially social media (102) guidelines, are growing in size and scope, seizing the opportunity posed by social media and new modes of communication for new types of engagement and support.

Third, suicide prediction using novel research and clinical approaches, such as machine learning methods and computerized adaptive testing, may advance accurate identification of persons at high risk of suicide (40, 42, 129). Knowledge of who has a high suicide risk can inform strategies for targeting evidence-based preventive interventions such as limiting access to lethal means or psychosocial interventions (17, 20, 70).

Reducing suicide mortality risk in the United States is a largely unmet public health need with a far-reaching and long-lasting impact that is increasing among vulnerable and minoritized population groups. Strategies to reduce suicide mortality risk in the United States should prioritize implementation and scale-up of recent advances in suicide prevention, with a focus on the social, economic, and political contexts where suicide risk unfolds as the result of life course adversity faced by vulnerable and minoritized groups.
